# Syndrome des ecchymoses douloureuses: une entité rare à ne pas méconnaitre

**DOI:** 10.11604/pamj.2014.17.73.3868

**Published:** 2014-01-30

**Authors:** Wafa Chebbi, Baha Zantour

**Affiliations:** 1 Service de Médecine Interne, CHU Taher Sfar Mahdia, Mahdia, Tunisie

**Keywords:** Syndrome, ecchymoses douloureuses, purpura psychogène, syndrome, painful bruising, psychogenic purpura

## Image en medicine

Le syndrome des ecchymoses douloureuses est une entité rare décrite pour la première fois par Gardner et Diamond en 1955. Il est caractérisé par l'apparition spontanée, chez des jeunes femmes au profil psychologique pathologique, d'ecchymoses douloureuses, siégeant le plus souvent aux extrémités, et évoluant par poussées, sans anomalies biologiques associées. Il s'y associe souvent diverses plaintes à type de céphalée, vertiges, paresthésies des extrémités, troubles digestifs, arthralgies et myalgies. Cet ensemble de signes cliniques peut simuler une maladie systémique, une dermohypodermite bactérienne ou un syndrome des loges. Sa prise en charge doit être globale, à la fois psychologique et somatique. Nous rapportons l'observation d'une patiente âgée de 20 ans qui consultait pour des lésions ecchymotiques du membre supérieur gauche récidivantes, d'apparition spontanée, accompagnées d'une sensation de brûlure et des arthralgies des épaules. L'examen clinique ne révélait aucune anomalie, à l'exception des ecchymoses. Le bilan biologique (numération formule sanguine, vitesse de sédimentation, protéine C-réactive, dosage des facteurs de la coagulation) était normal. La radiographie des épaules et l'angio-scanner du membre supérieur gauche à la recherche d'une cause compressive étaient normaux. Ces manifestations cutanées, sans anomalies biologiques associées, faisaient évoquer un syndrome des ecchymoses douloureuses. L’évaluation psychiatrique révélait une anxiété généralisée selon la définition DSM-IV avec des troubles du sommeil et des problèmes de concentration. Un traitement associant une psychothérapie de soutien et des antidépresseurs sérotoninergiques avait entraîné une régression des manifestations cutanées et des arthralgies. Aucune récidive des ecchymoses n’était observée après un recul de 3 ans.

**Figure 1:**
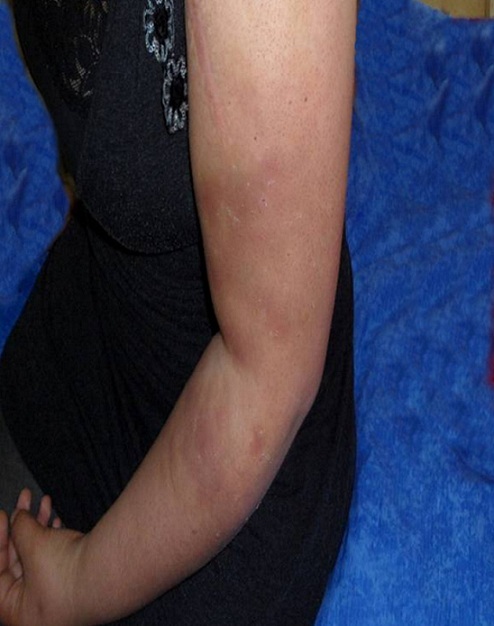
Lésions ecchymotiques du membre supérieur gauche

